# Incidence, origins and avoidable harm of missed opportunities in diagnosis: longitudinal patient record review in 21 English general practices

**DOI:** 10.1136/bmjqs-2020-012594

**Published:** 2021-06-14

**Authors:** Sudeh Cheraghi-Sohi, Fiona Holland, Hardeep Singh, Avril Danczak, Aneez Esmail, Rebecca Lauren Morris, Nicola Small, Richard Williams, Carl de Wet, Stephen M Campbell, David Reeves

**Affiliations:** 1 NIHR Greater Manchester Patient Safety Translational Research Centre, The University of Manchester Faculty of Biology, Medicine and Health, Manchester, UK; 2 NIHR School for Primary Care Research, Manchester Academic Health Science Centre, The University of Manchester Faculty of Biology, Medicine and Health, Manchester, UK; 3 Centre for Biostatistics, Manchester Academic Health Science Centre, The University of Manchester, Manchester, UK; 4 Center for Innovations in Quality, Effectiveness and Safety, Michael E. DeBakey Veterans Affairs Medical Center and Baylor College of Medicine, Houston, Texas, USA; 5 Central and South Manchester Specialty Training Programme for General Practice, Health Education England North West, Manchester, UK; 6 School of Medicine, Griffith University Faculty of Health, Gold Coast, Queensland, Australia

**Keywords:** general practice, patient safety, adverse events, epidemiology and detection, diagnostic errors

## Abstract

**Background:**

Diagnostic error is a global patient safety priority.

**Objectives:**

To estimate the incidence, origins and avoidable harm of diagnostic errors in English general practice. Diagnostic errors were defined as missed opportunities to make a correct or timely diagnosis based on the evidence available (missed diagnostic opportunities, MDOs).

**Method:**

Retrospective medical record reviews identified MDOs in 21 general practices. In each practice, two trained general practitioner reviewers independently conducted case note reviews on 100 randomly selected adult consultations performed during 2013–2014. Consultations where either reviewer identified an MDO were jointly reviewed.

**Results:**

Across 2057 unique consultations, reviewers agreed that an MDO was possible, likely or certain in 89 cases or 4.3% (95% CI 3.6% to 5.2%) of reviewed consultations. Inter-reviewer agreement was higher than most comparable studies (Fleiss’ kappa=0.63). Sixty-four MDOs (72%) had two or more contributing process breakdowns. Breakdowns involved problems in the patient–practitioner encounter such as history taking, examination or ordering tests (main or secondary factor in 61 (68%) cases), performance and interpretation of diagnostic tests (31; 35%) and follow-up and tracking of diagnostic information (43; 48%). 37% of MDOs were rated as resulting in moderate to severe avoidable patient harm.

**Conclusions:**

Although MDOs occurred in fewer than 5% of the investigated consultations, the high numbers of primary care contacts nationally suggest that several million patients are potentially at risk of avoidable harm from MDOs each year. Causes of MDOs were frequently multifactorial, suggesting the need for development and evaluation of multipronged interventions, along with policy changes to support them.

## Introduction

Diagnostic errors in primary care have harmful consequences for patients/caregivers, practitioners and health systems.^1^ Preventable harm may occur when undiagnosed conditions remain untreated or when patients undergo unnecessary (or harmful) tests. Primary care underpins the UK health system, with an estimated 340 million patient consultations in England annually (or approximately 85% of all National Health Service doctor–patient encounters).^2^ Diagnostic errors account for the greatest proportion of malpractice claims against family practitioners (general practitioners, GPs) in the UK (63%) incurring multiple costs.^3^ The WHO^4^ and the National Academy of Medicine^5^ have identified measuring and reducing diagnostic error as a patient safety priority.^4^

Judging the magnitude of diagnostic errors requires accurate and reliable estimation. The scale and cause(s) of such errors will vary across countries and contexts. Diagnostic error is estimated to involve 5% of US adults in the outpatient setting or approximately 12 million adults annually.^6^ A recent study produced the first estimate for the incidence of ‘avoidable significant harm’ in UK primary care at 35.6 per 100 000 patient-years.^7^ However, that rate includes all causes of avoidable harm, with diagnostic error accounting for around 60%. Using an established definition of diagnostic error, defined as missed opportunities to make a correct or timely diagnosis based on the evidence available, the current study estimates the incidence, origins and avoidable harms of ‘missed diagnostic opportunities’ (MDOs) in UK primary care.

## Methods

### Conceptual approach to measurement

Diagnostic errors include missed, delayed or wrong diagnosis and how they are defined is a central issue for measurement.^8^ One established approach defines diagnostic error only when there is clear *evidence* of a ‘missed opportunity’ to make a correct or more timely diagnosis: that is, something different could have been done within the context of an evolving diagnostic process. In this study, we used this approach to identify ‘missed diagnostic opportunities’ and excluded clinical situations with no evidence for an opportunity to intervene (see online supplemental figure S1).^9 10^

10.1136/bmjqs-2020-012594.supp1 Supplementary data

The aetiology of MDOs is multifactorial arising via a complex interplay of contributory factors.^11^ To characterise origins and potential contributory factors, we used the ‘*Safer Dx*’ conceptual framework which accounts for breakdowns in the processes of care related to diagnosis.^12^ This framework involves five interactive process dimensions that could be involved in the breakdown: (1) the patient–provider encounter (history, physical examination, ordering tests/referrals based on assessment); (2) performance and interpretation of diagnostic tests; (3) follow-up and tracking of diagnostic information over time; (4) subspecialty and referral-specific factors; and (5) patient-related factors. Multiple dimensions may be involved in any particular MDO and the framework is designed to address both cognitive and system-related factors.

The incidence of diagnostic errors has been estimated using different methods,^9^ each with strengths and weaknesses.^13^ Retrospective patient record reviews, focused on the care provided across a sample of ‘index consultations’, are a widely used approach,^14^ and in the context of primary care allow for diagnostic information and process to be traced over time, often across multiple consultations and different settings of care. But error rates based on single-reviewer assessments may be of insufficient validity and the use of at least two independent reviewers is recommended.^15^ Even with two reviewers, reported rates of inter-reviewer reliability have often been low.^16–18^

In this study, all included index consultations and related case notes in the electronic health records (EHRs) were first assessed by two reviewers acting independently. In addition to providing data for calculating inter-reviewer agreement and confirmation of identified MDOs, this also reduced the risk of any MDOs being missed. Next, all consultations that one or both reviewers regarded as involving an MDO were reassessed by the two reviewers working together. Rather than obligate reviewers to arrive at a consensus, we allowed them the option to continue to disagree.

### Sample

#### Practices

We originally planned a two-phase practice recruitment and record review process.^19^ Phase 1 aimed to calibrate reviewer performance in identifying and assessing MDOs through the use of ‘double’ reviews of records at 15 GP practices, while phase 2 involved further data collection at upwards of 35 additional practices using single reviewers. However, due to recruitment delays and resource constraints we decided to conduct only phase 1. We observed less clustering of MDOs within practices than the protocol sample size calculations had assumed, and calculated that a final sample of 21 practices would estimate the overall MDO rate within 95% error limits of ±2% at most. We performed double reviewing at all 21 practices.

GP practices were recruited through face-to-face meetings, letters and word of mouth. While based in North West England, they were selected to be nationally representative in practice size and area deprivation. Practice size was coded in national quintiles of registered patient numbers and area deprivation in national quintiles of the 2010 English Index of Multiple Deprivation (IMD) according to practice postcode. The English IMD is a UK Government summary measure across seven domains of deprivation (income, employment, education, skills and training, health and disability, crime, barriers to housing and services and living environment),^20^ and is associated with area healthcare need. The sample was broadly representative of national practice populations, excepting some under-representation of highly affluent areas resulting from regional demographics (online supplemental table S1). Study practices had a median 2013/2014 English Quality and Outcomes Framework overall score (a UK Government-derived measure of quality of English primary care) close to the national median (96.5% vs 96.2%), with the lowest scoring practice at the national 9th percentile.

#### Patients

Reasons for consulting in primary care vary across the year,^7^ therefore a random sampling frame was developed to ensure sampled consultations were spread evenly across the calendar year, both within and across practices. Each practice was assigned four 1-week periods within which consultations were sampled, separated by 13-week intervals, ranging from 8 July 2013 to 8 December 2014. Starting dates were chosen such that out of 84 sampling weeks in total, 7 were from each calendar month (ie, 7 from January, 7 from February, etc). For each sampling week, a random sample of 25 face-to-face consultations by different eligible patients was selected. All patients included were aged 18 years or more at the start of the study review period and were registered with the practice from at least 3 months prior to the consultation to at least 9 months after. Thus, 100 index consultations per practice, along with associated case notes available in the EHR, were selected for review across 1 year.

### Data collection

A two-stage approach was adopted (see online supplemental figure S2), as detailed previously.^19^ At stage 1, a project administrator visited each practice to collect data on an electronic data collection instrument detailing the selected patients’ demographics (sex, age) and basic health information (number of long-term conditions, number of repeat medications, location of the index consultation).

At stage 2, retrospective clinical review of the EHR case notes was undertaken by trained GPs. Four GPs with experience ranging from 2 to 42 years (average 19 years) visited the practices or remotely accessed records to conduct the reviews. Each index consultation in all 21 practices was assessed by two reviewers. The first reviewer, with more than 30 years’ experience as a GP, was the same individual for all practices (GP 1); the second reviewer was one of the three remaining GP reviewers. Reviewer 1 conducted case reviews at all 21 practices, while reviewer 2 evaluated the same sets of records at 16 of these, and reviewers 3 and 4 at 3 and 2 practices respectively (see online supplemental table S2). All reviewers received rigorous hands-on training and pilot tested the review process over multiple sessions.

A reviewer first assessed each index consultation for the presence of any new diagnostic activity. Where such activity was detected a full review was conducted, otherwise the review was halted and the reviewer moved on to the next randomly selected patient. For each review, all relevant extracted information, decisions made and supporting free text notes were entered into an electronic case report form (CRF). Under a full review, the content of the case notes was assessed for a time window of at least 3 months prior to the index consultation to a minimum of 9 afterwards, to allow the patient’s diagnostic journey to be traced. Based on previous experience, this time frame was expected to capture all the main relevant evidence and allow large numbers of reviews to be completed within the study period.^18^ However, to clarify some MDOs and their impact, reviewers could extend the time frame if deemed necessary. The data collection instrument guided the reviewer through the record in a systematic and structured manner, to form a professional judgement on the accuracy of each new diagnosis. The process was informed by two related tasks: (1) searching for the presence, frequency and reason(s) for six predefined ‘prompts’ potentially indicative of MDOs (ie, consultations, referrals, hospital admissions, out of hours contacts, accident and emergency attendance, imaging requests); and (2) consideration of evidence in response to a set of eight questions adapted from an earlier version of the Safer Dx instrument,^21^ a tool for evaluating primary care record reviews for MDOs, which was under development at that time in the USA (see online supplemental box S1). At the end of this process the reviewer coded each new diagnosis into one of six categories (see online supplemental box S2 and online supplemental table S2): (1) diagnosis accurate and sufficient evidence to make a confident determination of accuracy; (2) diagnosis accurate but insufficient evidence to make a confident determination; (3) diagnosis accurate but incomplete and/or little or no evidence; (4) MDO possible (professional interpretation but little or no documented evidence); (5) MDO likely (at least some documented evidence); and (6) MDO certain (compelling documented evidence). To help understand the origins of each MDO, reviewers were asked to determine the corresponding breakdown process(es) involved. A corresponding rating of the harm attributable to each MDO was obtained using a 5-point scale, adapted from previously published work^22^ on the impact of adverse effects in family practice: ‘no harm’, ‘mild harm’, ‘moderate harm’, ‘severe harm’ and ‘unclear’. Reviewers assessed the actual harm as evidenced in the case notes and were guided by the definitions and examples provided in the study manual (online supplemental box S3). For example, mild harms included minor or inconvenient impacts without any residual effect, such as unnecessary appointments; moderate harms were those that caused prolonged distress or impact, for example, prolonged distress resulting in unnecessary sick leave; severe harms involved consequences with prolonged or permanent impact, for example, preventable hospital admission/procedures/complications, disease progression, disability or death. MDOs where the reviewer was unable to make a determination were given an ‘unclear’ rating.

The procedures by which reviews were conducted were designed to also promote calibration of reviewers’ rating behaviour over time. At each practice, the two reviewers first independently reviewed case notes of 50 patients from the practice. All cases where at least one reviewer identified an MDO were then jointly discussed and an agreed final determination was sought; however, agreement was not mandatory. Cases where neither reviewer identified an MDO were not jointly reviewed. This procedure was then repeated at a later date for the practice’s second set of 50 patients.

### Data analysis

When assessing the same index consultation, reviewers could and did differ in the number and types of new diagnoses they identified, making it impossible to compare reviewer ratings at the level of individual diagnoses within a patient. The unit of analysis was therefore the consultation and not the diagnosis. Only 8.5% of all reviews identified more than one new diagnosis. Therefore, where a reviewer made multiple diagnoses, we selected the one with the highest MDO rating along with the corresponding harm rating.

Following the approach used by previous studies,^13^ for the main analysis we collapsed the ratings made using the initial fine-grained coding scheme, together with ratings of ‘no new diagnosis’, into three main groupings: (1) no new diagnosis; (2) a new and accurate diagnosis was present (combining sufficient, insufficient and incomplete/no evidence); and (3) an MDO was implicated (combining possible, likely and certain MDOs). However, findings reporting the full 6-point scale are presented where appropriate.

#### Calculating reviewer agreement

We calculated rates of agreement within each main grouping (no new diagnosis; new accurate diagnosis; MDO) between the first and second reviewers separately for the ratings made independently and jointly. We also computed agreement separately for the records assessed in the first and second 50% of review sessions, within each pair of reviewers. Agreement was computed as the percentage of consultations where both reviewers gave the same rating, out of the total number where either or both gave that rating. We used the STATA V.15 user-written ‘kapci’ command to compute Fleiss’ kappa (interpreted in the same way as Cohen’s kappa) accounting for the use of three different pairs of reviewers and to estimate CIs using a non-parametric bootstrap method.

#### Calculating rates of MDOs

We considered an MDO to be present in an index consultation if, after joint review, the two reviewers agreed that an MDO was implicated as ‘possible’, ‘likely’ or ‘certain’. Consultations where the reviewers disagreed on the presence of an MDO—despite having reviewed the evidence together—were not considered to involve an MDO. We computed the incidence of confirmed MDOs as a percentage of all index consultations. We also report the number of MDOs mutually agreed to be ‘likely’ or ‘certain’ after joint review: these represent cases for which both reviewers found stronger documented evidence to exist in the record, and hence for which certainty is greatest.

The STATA ‘proportion’ command was used to estimate proportions and compute the corresponding 95% CI accounting for clustering of observations within practices. We used descriptive statistics to summarise the characteristics of the identified MDOs, including the medical conditions and breakdown processes involved. Univariate and multivariate logistic regression was applied to investigate associations between MDOs and patient gender, age, number of repeat medications, number of long-term conditions and consultation location (practice or at home). All but gender and location were treated as continuous variables and practice as a random effect.

## Results

A total of 2100 individual consultations were sampled. Seven were not examined due to administrative error and 29 were subsequently found to be ineligible due to: the patient being under 18 years (22 records); not a face-to-face consultation (2); insufficient follow-up (1); other reason (4). The resulting 2064 consultations related to a patient sample with a mean age of 49.5 years (median 49; IQR 34–64; 18% ≥70), that was 41% male with a mean of 2.8 long-term conditions and 2.8 repeat medications.

### Independent reviewer assessments

Table 1 shows that acting independently, the first and second reviewers rated reasonably similar percentages of index consultations as including no new diagnosis (45.2% vs 51.1%), a new and accurate diagnosis (48.2% and 43.8%) or as implicating an MDO (6.6% and 5.1%).

**Table 1:** Summary of number of practices visited and results of independent case reviews, by first reviewer and second* reviewer

Reviewer	Number of practices visited	Number of case reviews conducted	No new diagnosis	New, accurate diagnosis	MDO implicated
First reviewer	21	2064	933 (45.2%)	995 (48.2%)	136 (6.6%)
Second reviewer	21	2064	1055 (51.1%)	903 (43.8%)	106 (5.1%)

*’Second’ reviewer refers to reviewers 2, 3 and 4 combined.

MDO, missed diagnostic opportunity.

### Inter-reviewer agreement

Results for the built-in calibration exercise were mixed (online supplemental table S3). Between the first and second 50% of independent review sessions overall agreement increased from 75.6% (776/1027) to 83.3% (864/1037): however, while agreement that a consultation did not contain an MDO increased from 62.4% (370/593) to 71.6% (390/545), agreement on an MDO dropped from 19.8% (23/166) to 13.2% (12/91), though the numbers involved were small. We therefore based all subsequent analysis on the full set of 2064 consultations.

Overall agreement across all 2064 records was 79.4% (1640/2064) and the kappa coefficient was 0.63 (95% CI 0.60 to 0.66) (table 2). However, reviewers were more likely to agree on the absence of an MDO (66.8%; 760/1138) than on its presence (16.9%; 35/207); a pattern observed in other error rate studies.^23 24^

**Table 2:** Summary of reviewer rates of agreement for the independent and joint reviews (number of index consultations rated as such by both reviewers/number rated as such by either; % agreement)

	No new diagnosis	New accurate diagnosis (no MDO)	MDO implicated	Overall agreement	Kappa* (95% CI)
Independent reviews (n=2064)	845/1143 (73.9%)	760/1138 (66.8%)	35/207† (16.9%)	1640/2064 (79.4%)	0.63 (0.60 to 0.66)
Joint reviews (n=2057‡)	858/1121 (76.5%)	835/1106 (75.5%)	89/105† (84.8%)	1782/2057 (86.6%)	Not relevant

*Kappa 95% CI estimated using 1000 bootstrap samples.

†Indicates that when reviewing independently, 35 consultations were coded as including an MDO by both reviewers, while another 172 (total=207) were coded as an MDO by one reviewer but as no new diagnosis or a new accurate diagnosis by the other; resulting in 16.9% agreement (35/207×100%). After the joint reviews 89 consultations were agreed by the reviewers to include an MDO, with another 16 (total=105) coded an MDO by one but not the other; giving 84.8% agreement.

‡Excludes seven index consultations unintentionally omitted from the joint review exercise.

MDO, missed diagnostic opportunity.

Of the 207 consultations identified by at least one reviewer as implicating an MDO, 200 were subjected to joint review, with seven omitted due to administrative oversight. In addition, for 56 jointly reviewed consultations, the agreed determination failed to be recorded in the CRF at the time. For these consultations the lead reviewer extracted a final determination from the reviewers and observer’s notes of the meeting.

After the joint reviews, agreement on the presence of an MDO increased to 84.8% (89/105). Table 3 provides a cross-tabulation of the joint review ratings at the fine-grained level. Of 16 consultations where the reviewers could not agree if an MDO was present or not, in 15 instances one reviewer rated the consultation a possible MDO only, while the other coded it as no MDO or no new diagnosis.

**Table 3:** Cross-tabulation of first reviewer and second reviewer classifications of index consultations after the joint reviews

First reviewer	Second reviewer							
	No new diagnosis	New, accurate diagnosis, with sufficient evidence	New, accurate diagnosis, with insufficient evidence	New, accurate diagnosis, with incomplete evidence	Missed diagnostic opportunity possible	Missed diagnostic opportunity likely	Missed diagnostic opportunity certain	Total
No new diagnosis	858	36	23	15	0	0	0	932
New, accurate diagnosis, with sufficient evidence	18	64	18	4	0	0	0	104
New, accurate diagnosis, with insufficient evidence	41	146	96	37	0	0	0	320
New, accurate diagnosis, with incomplete evidence	126	176	164	130	2	0	1	599
Missed diagnostic opportunity possible	4	4	1	4	40	8	7	68
Missed diagnostic opportunity likely	0	0	0	0	2	18	7	27
Missed diagnostic opportunity certain	0	0	0	0	0	0	7	7
Total	1047	426	302	190	44	26	22	2057

### MDO incidence

After joint review, reviewers agreed that an MDO was implicated in 89 of the 2057 index consultations, giving an overall MDO rate of 4.3% (95% CI 3.6% to 5.2%). Because new diagnostic activity was identified (by at least one reviewer) in only 1199 index consultations (58%), we calculated the MDO rate for this set separately and found it to be 7.4%. Of the 89 MDOs, 32 were jointly assessed as likely or certain, representing the cases for which the reviewers agreed the evidence was strongest.

Because of the risk that the 56 final determinations made by the lead reviewer working from the joint review notes could be of lower reliability, we assessed this for potential impact. After recomputing the MDO rate using each reviewer’s independent rating for these cases, we found only a small change in the number of confirmed MDOs, which reduced from 89 (4.3%) to 80 (3.9%).

### Characteristics of the MDOs

The identified MDOs are summarised in table 4: nearly 50% involved diseases of the genitourinary system, skin/subcutaneous tissue, digestive system; and endocrine, nutritional and metabolic diseases. Online supplemental box S4 provides specific examples of some of the MDOs, along with associated levels and types of harm.

**Table 4:** Summary of the missed diagnoses identified after joint review

ICD-10 chapter	Count
Diseases of the genitourinary system	11
Diseases of the skin and subcutaneous tissue	11
Diseases of the digestive system	9
Endocrine, nutritional and metabolic diseases	9
Diseases of the circulatory system	8
Diseases of the respiratory system	8
Mental and behavioural disorders	8
Neoplasms	5
External causes of morbidity and mortality	3
Medication side effects	3
Symptoms, signs and abnormal clinical and laboratory findings	3
Certain infectious and parasitic diseases	2
Diseases of the blood	2
Diseases of the musculoskeletal system and connective tissue	2
Diseases of the nervous system	2
Diseases of the ear and mastoid process	1
Pregnancy, childbirth and the puerperium	1
Symptoms and signs involving the skin and subcutaneous tissue	1
Total	89

ICD-10, International Classification of Diseases, 10th Revision.

The most common factors contributing to MDOs (table 5) were problems within the patient–practitioner encounter (such as history, physical examination and ordering of diagnostic tests), which was the main contributing factor in 52 (58%) cases and a secondary factor in another 9 (10%). This was followed by performance and interpretation of diagnostic tests, which was a main factor in 22 (25%) and a secondary factor in 9 (10%), and issues relating to follow-up and tracking of diagnostic information, a main factor in 21 (24%) and secondary factor in 22 (24%). Sixty-four (72%) of the identified MDOs were judged to have two or more process breakdowns (online supplemental table S4).

**Table 5:** Number (%) of reviewer-assessed contributing factors to the MDOs

Contributing factor	MDOs with this as the lead contributing factor n (%*)	MDOs with this as the lead or a secondary contributing factor n (%*)
Patient–practitioner clinical encounter	52 (58)	61 (68)
Performance and/or interpretation of diagnostic tests	22 (25)	31 (35)
Follow-up and tracking of diagnostic information	21 (24)	43 (48)
Subspecialty and referral related	11 (12)	17 (19)
Patient-specific processes	5 (6)	14 (16)
Unclear	1 (1)	3 (3)

Percentages add to >100% due to multiple contributing factors for each MDO.

*Denominator is index consultations with at least one MDO, n=89.

MDO, missed diagnostic opportunity.

Table 6 summarises associations between the presence of an MDO and available patient characteristics. Consultation location was excluded due to low variation. P values varied considerably under the univariate and multivariate models as a result of high intercorrelations and no consistently significant relationships were found.

**Table 6:** Summary of logistic regression relating patient characteristics to the occurrence of an MDO

	Patients with a confirmed MDO (n=89)	Patients with no confirmed MDO (n=1961)*	Univariate analysis		Multivariate analysis		
			OR (95% CI)	P value	OR (95% CI)	P value	Sensitivity p value†
Age, mean (SD)	52.0 (17.4)	49.4 (18.3)	1.08‡ (0.98 to 1.19)	0.112	1.11‡ (0.99 to 1.26)	0.077	0.022
Male, n (%)	39 (44)	801 (41)	1.13 (0.85 to 1.51)	0.407	1.12 (0.83 to 1.50)	0.458	0.479
Repeat medications, mean (SD)	2.7 (3.5)	2.8 (3.6)	0.97 (0.94 to 1.04)	0.601	0.94 (0.88 to 1.00)	0.056	0.131
Long-term conditions, mean (SD)	2.9 (2.5)	2.8 (2.3)	1.03 (0.93 to 1.14)	0.564	1.04 (0.92 to 1.17)	0.548	–
Consultation location, n (%)§							
Practice	88 (99)	1936 (99)	NA	NA	NA	NA	NA
Home	1 (1)	18 (1)					

*Seven patients excluded due to missing covariate data.

†Excluding number of long-term conditions due to high multicollinearity (r=0.63 with number of medications; r=0.55 with age).

‡OR for a 10-year change in age.

§Not included in logistic model because only one patient with an MDO was seen at home.

MDO, missed diagnostic opportunity; NA, not applicable.

### Harm ratings

Thirty-three (37.1%) of the MDOs were rated as having caused moderate or severe patient harm, based on the highest rating from each reviewer pair (online supplemental table S5), implying a significant degree of physical or psychological distress and possibly prolonged or permanent consequences. Another 47 (52.8%) caused only mild harm, mostly patient inconvenience. Very few MDOs were assessed to have caused no harm (5; 5.6%) or to be unclear in their impact (4; 4.5%).

## Discussion

Reviewer pairs agreed that an MDO was implicated in 89 index consultations (4.3%). Of these, 33 (37%) were assessed to have caused moderate or severe patient harm. Overall rates of inter-reviewer agreement and the kappa value were considerably higher than has been reported by most other diagnostic error studies.^15 16 18 23^ Our findings add to the growing evidence of the burden of diagnostic error in the UK. Across 12 randomly invited practices, Avery *et al* 7 recently found 74 instances of avoidable harm among 2131 UK consultations with a significant new health problem, of which 45 were due to diagnostic errors (wrong or delayed diagnosis), suggesting a diagnostic error rate of 2.1%. A comparable figure from our study—based in a different health region and using a different study design, error definition and approach to measurement—is the 33 MDOs associated with more than just mild harm out of 1199 consultations with new diagnostic activity, 2.8%. Our results are also comparable to US estimates of diagnostic errors in outpatient care.^18^ While more than 95% of consultations in our study did not involve an MDO, there are an estimated 340 million consultations in UK general practice annually.^25^ This suggests that should the MDO rate of 4.3% in our practice sample be replicated nationally, there could be up to 15 million MDOs nationally, with up to 6 million associated with potentially avoidable moderate to severe patient harm.

Given the complexity of general practice, estimating diagnostic errors in primary care is challenging.^26^ GPs are often tasked with identifying patients with a serious disease from large numbers who present with common symptoms and mostly benign non-urgent diseases, many of which evolve over time.^27^ A significant proportion of face-to-face consultations are for reasons other than making a diagnosis. Nonetheless, although reviewers often differed about fine-grained details such as levels of available evidence and degrees of confidence that an MDO had occurred, joint review agreement was high regarding the essential question of whether or not a consultation included a missed opportunity to make a diagnosis.

Although the vast majority of GP consultations occur without incident, errors occur for a wide variety of reasons and many missed opportunities had multifactorial cognitive and system origins. Poor communication and care coordination between healthcare professionals and difficulties in doctor–patient communication occurred similar to prior studies.^28^ Several factors were beyond the control of the clinician (eg, system or patient factors),^29^ such as when patients did not attend appointments for tests or referrals and premature discharge from secondary care. The risk of error may increase with increasing workload pressures,^25^ patient care across multiple settings of care, resource-constrained work environments and complexity of patients.^30^ Physician stress and associated burn-out can also be associated with increased medical error,^31^ as can GPs not looking beyond the most obvious diagnosis or not considering atypical presentations.^32^ The COVID-19 pandemic and associated pressures on the health system are likely to increase the risk of MDO occurrence for many reasons,^33^ and have already been shown to have an impact in the UK on many conditions commonly seen in primary care.^34^ Understanding the reasons for MDOs and monitoring their occurrence should be a priority for the UK health system going forward.

We used a representative sample of practices and large sample of patients randomly selected from adult face-to-face consultations across a 1-year period. All records were assessed for MDOs by two trained reviewers working both independently and jointly, to minimise the risk of underidentification and provide confirmation of MDOs. Despite building a calibration process into the design, the impact on inter-reviewer agreement when working independently was mixed. Nonetheless, inter-reviewer agreement and the kappa statistic were higher than most comparable studies of diagnostic error and the large majority of disagreements were resolved by joint review.

There were several study limitations. Our practice sample was restricted to North West England. However, our results compare well with those of Avery *et al* using practices from three other UK health regions. The case notes reviewed related mostly to care provided in 2014 and 2015 (including follow-up), and so may not fully reflect current diagnostic activity or rates of MDOs. However, we are unaware of any specific changes in daily practice that would have influenced our findings had we done this study more recently, for example in 2019 (before the pandemic). Future studies should nevertheless evaluate more recent patterns. Our sampling frame purposely excluded patients with less than 9 months of follow-up, allowing sufficient time for harmful consequences to emerge and be identified, but as a result MDOs of moderate/severe harm leading to early practice transfer-out or death may be under-represented. While retrospective methods are susceptible to hindsight/observer bias in judgements about error determination,^35^ they provide opportunities to review longitudinal data that reflect the evolution of the diagnostic process culminating in a final diagnosis.^17^ The judgement of whether an MDO occurred is based on evidence contained in the case notes minimising subjective interpretation; nevertheless, variations in documentation and coding across practices make this assessment complex, and subject to reviewer judgements. Keeping one reviewer the same for all reviewed consultations may have increased consistency of assessments, but may have also induced some bias towards that reviewer’s subjective viewpoints. Information on mitigating factors and other contextual information are often missing from case notes, making it difficult to determine what the clinician was thinking at the time of the diagnosis. Finally, ratings of harm severity were based on available evidence over the time span of the reviewed case notes, which may have failed to pick up the longer term impacts of some MDOs. Hence, in some cases, the true extent of harm may have been underestimated.

## Conclusion

MDOs occurred in fewer than 5% of the investigated consultations. Nonetheless, the high numbers of primary care contacts nationally suggest that several million UK patients are potentially at risk of avoidable harm each year due to MDOs. Causes of MDOs are frequently multifactorial suggesting the need for multipronged interventions including policy changes that support them. Understanding the reasons for MDOs and monitoring their occurrence should be a priority for all health systems going forward.

## Data Availability

Data not included in the article or uploaded as supplementary information is available upon reasonable request.

## References

[1] Panesar SS , deSilva D , Carson-Stevens A , et al . How safe is primary care? A systematic review. BMJ Qual Saf 2016;25:544–53. 10.1136/bmjqs-2015-004178 26715764

[2] England N . General practice forward view, 2016.

[3] Newman-Toker DE , McDonald KM , Meltzer DO . How much diagnostic safety can we afford, and how should we decide? A health economics perspective. BMJ Qual Saf 2013;22 Suppl 2:ii11–20. 10.1136/bmjqs-2012-001616 PMC3786645 24048914

[4] Cresswell KM , Panesar SS , Salvilla SA , et al . Global research priorities to better understand the burden of iatrogenic harm in primary care: an international Delphi exercise. PLoS Med 2013;10:e1001554. 10.1371/journal.pmed.1001554 24260028 PMC3833831

[5] National Academies of Sciences E, and Medicine . Improving diagnosis in health care. Washington DC, 2015.

[6] Singh H , Meyer AND , Thomas EJ . The frequency of diagnostic errors in outpatient care: estimations from three large observational studies involving us adult populations. BMJ Qual Saf 2014;23:727-31. 10.1136/bmjqs-2013-002627 PMC4145460 24742777

[7] Avery AJ , Sheehan C , Bell B , et al . Incidence, nature and causes of avoidable significant harm in primary care in England: retrospective case note review. BMJ Quality &amp;amp; Safety 2021;30:961–76. 10.1136/bmjqs-2020-011405 PMC8606464 33172907

[8] Graber ML , Franklin N , Gordon R . Diagnostic error in internal medicine. Archives of Internal Medicine 2005;165:1493–9. 16009864 10.1001/archinte.165.13.1493

[9] Singh H , Schiff GD , Graber ML , et al . The global burden of diagnostic errors in primary care. BMJ Qual Saf 2017;26:484–94. 10.1136/bmjqs-2016-005401 PMC5502242 27530239

[10] Singh H . Editorial: helping health care organizations to define diagnostic errors as missed opportunities in diagnosis. The Joint Commission Journal on Quality and Patient Safety 2014;40:99–AP1. 24730204 10.1016/s1553-7250(14)40012-6

[11] Graber ML . The incidence of diagnostic error in medicine. BMJ Qual Saf 2013;22:ii21–7. 10.1136/bmjqs-2012-001615 23771902 PMC3786666

[12] Singh H , Sittig DF . Advancing the science of measurement of diagnostic errors in healthcare: the safer DX framework. BMJ Qual Saf 2015;24:103–10. 10.1136/bmjqs-2014-003675 PMC4316850 25589094

[13] Thomas EJ , Studdert DM , Burstin HR . Incidence and types of adverse events and negligent care in Utah and Colorado. Med Care 2000;38:261–71. 10718351 10.1097/00005650-200003000-00003

[14] Zwaan L , Schiff GD , Singh H . Advancing the research agenda for diagnostic error reduction. BMJ Quality & Safety 2013;22:ii52–7. 10.1136/bmjqs-2012-001624 PMC3786655 23942182

[15] Forster AJ , O'Rourke K , Shojania KG . Combining ratings from multiple physician reviewers helped to overcome the uncertainty associated with adverse event classification. Journal of Clinical Epidemiology 2007;60:892–901. 17689805 10.1016/j.jclinepi.2006.11.019

[16] Gunderson CG , Bilan VP , Holleck JL , et al . Prevalence of harmful diagnostic errors in hospitalised adults: a systematic review and meta-analysis. BMJ Qual Saf 2020;29:1008–18. 10.1136/bmjqs-2019-010822 32269070

[17] Zwaan L , de Bruijne M , Wagner C . Patient record review of the incidence, consequences, and causes of diagnostic adverse events. Arch Intern Med 2010;170:1015–21. 20585065 10.1001/archinternmed.2010.146

[18] Singh H , Giardina TD , Forjuoh SN . Electronic health record-based surveillance of diagnostic errors in primary care. BMJ Quality and Safety 2012;21:93. 10.1136/bmjqs-2011-000304 PMC3680372 21997348

[19] Cheraghi-Sohi S , Singh H , Reeves D . Missed diagnostic opportunities and English general practice: a study to determine their incidence, confounding and contributing factors and potential impact on patients through retrospective review of electronic medical records. Implementation Science 2015;10:105. 26220545 10.1186/s13012-015-0296-z PMC4518650

[20] Communities and Local Government . English indices of deprivation 2010: department for communities and local government, 2011. Available: https://assets.publishing.service.gov.uk/government/uploads/system/uploads/attachment_data/file/6222/1871538.pdf [Accessed 5th October 2020].

[21] Al-Mutairi A , Meyer AN , Thomas EJ . Accuracy of the safer DX instrument to identify diagnostic errors in primary care. Journal of general internal medicine 2016;31:602–8. 26902245 10.1007/s11606-016-3601-x PMC4870415

[22] Wetzels R , Wolters R , van Weel C . Harm caused by adverse events in primary care: a clinical observational study. J Eval Clin Pract 2009;15:323–7. 19335492 10.1111/j.1365-2753.2008.01005.x

[23] Zegers M , de Bruijne MC , Wagner C . The inter-rater agreement of retrospective assessments of adverse events does not improve with two reviewers per patient record. Journal of Clinical Epidemiology 2010;63:94–102. 19473812 10.1016/j.jclinepi.2009.03.004

[24] Localio AR , Weaver SL , Landis JR . Identifying adverse events caused by medical care: degree of physician agreement in a retrospective chart review. Annals of Internal Medicine 1996;125:457–64. 8779457 10.7326/0003-4819-125-6-199609150-00005

[25] Hobbs FDR , Bankhead C , Mukhtar T . Clinical workload in UK primary care: a retrospective analysis of 100 million consultations in England, 2007-14. The Lancet 2016;387:2323–30. 10.1016/S0140-6736(16)00620-6 PMC4899422 27059888

[26] Singh H , Carayon P . A roadmap to advance patient safety in ambulatory care. JAMA 2020;324:2481–2. 33351052 10.1001/jama.2020.18551 PMC8016440

[27] Zwaan L , Singh H . Diagnostic error in hospitals: finding forests not just the big trees. BMJ Qual Saf 2020;29:961–4. 10.1136/bmjqs-2020-011099 32753410

[28] Sandars J , Esmail A . The frequency and nature of medical error in primary care: understanding the diversity across studies. Family Practice 2003;20:231–6. 12738689 10.1093/fampra/cmg301

[29] Michel P , Brami J , Chanelière M . Patient safety incidents are common in primary care: a national prospective active incident reporting survey. Plos One 2017;12:e0165455. 28196076 10.1371/journal.pone.0165455 PMC5308773

[30] Baird BC , Honeyman A; , Maguire M . D & Das, P. Understanding pressures in general practice. London 2016.

[31] Shanafelt TD , Balch CM , Bechamps G . Burnout and medical errors among American surgeons. Annals of surgery 2010;251:995–1000. 19934755 10.1097/SLA.0b013e3181bfdab3

[32] Ely JW , Kaldjian LC , Alessandro DM . Diagnostic errors in primary care: lessons learned. The Journal of the American Board of Family Medicine 2012;25:87. 22218629 10.3122/jabfm.2012.01.110174

[33] Tejal K Gandhi M, mph, Singh H. reducing the risk of diagnostic error in the COVID-19 era. Journal of Hospital Medicine 2020;6:363–6. 10.12788/jhm.3461 PMC7289509 32490798

[34] Williams R , Jenkins DA , Ashcroft DM . Diagnosis of physical and mental health conditions in primary care during the COVID-19 pandemic: a retrospective cohort study. The Lancet Public Health 2020;5:e543–50. 32979305 10.1016/S2468-2667(20)30201-2 PMC7511209

[35] Zwaan L , Singh H . The challenges in defining and measuring diagnostic error. Diagnosis 2015;2:97–103. 26955512 10.1515/dx-2014-0069 PMC4779119

