# Supplementary material for: Incidence, origins and avoidable harm of missed opportunities in diagnosis: longitudinal patient record review in 21 English general practices
Source: BMJ Qual Saf. 2021 Jun 14;30(12):977–85. doi: 10.1136/bmjqs-2020-012594 (PMC8606447; doi:10.1136/bmjqs-2020-012594)
Supplement: Supplementary data [file bmjqs-2020-012594supp001.pdf]

Incidence, origins and avoidable harm of missed opportunities in diagnosis: longitudinal patient record review in 21 English general practices

Supplementary file

Table S1: Number of participating practices by list size and index of multiple deprivation quintiles

**List size quintile**	**Index of multiple deprivation 2010 quintile**					**Total**
	1 (least deprived)	2	3	4	5 (most deprived)	
1 (smallest)	0	1	0	1	1	3
2	0	0	1	2	1	4
3	0	0	2	1	3	6
4	0	1	0	1	1	3
5 (largest)	1	1	1	2	0	5
**Total**	1	3	4	7	6	21

Table S2: Summary of number of practices visited and results of independent case reviews, by reviewer

Reviewer	Number of practices visited	Number of case reviews conducted	No new diagnosis	New, accurate diagnosis				MDO implicated			
				Sufficient evidence	Insufficient evidence	Incomplete evidence	Combined	Possible	Likely	Certain	Combined
1	21	2064	933<br>45.2%	103<br>5.0%	319<br>15.5%	573<br>27.8%	995<br>48.2%	103<br>5.0%	27<br>1.3%	6<br>0.3%	136<br>6.6%
2	16*	1573	848<br>53.9%	331<br>21.0%	194<br>12.3%	114<br>7.2%	639<br>40.6%	47<br>3.0%	18<br>1.1%	21<br>1.3%	86<br>5.5%
3	3*	294	108<br>36.7%	84<br>28.6%	59<br>20.1%	32<br>10.9%	175<br>59.5%	9<br>3.1%	1<br>0.3%	1<br>0.3%	11<br>3.7%
4	2*	197	99<br>50.3%	22<br>11.2%	55<br>27.9%	12<br>6.1%	89<br>45.2%	8<br>4.1%	1<br>0.5%	0<br>0.0%	9<br>4.6%
2, 3, 4^	21	2064	1055<br>51.1%	437<br>21.2%	308<br>14.9%	158<br>7.7%	903<br>43.8%	64<br>3.1%	20<br>1.0%	22<br>1.1%	106<br>5.1%

MDO=Missed Diagnostic Opportunity
*Independent sub-sets of the 21 practices also visited by reviewer 1
^Reviewers 2, 3 and 4 combined.

Table S3: Summary of reviewer rates of agreement when acting independently, for first and second 50%^ of review sessions (number of index consultations rated as such by both reviewers/number rated as such by either; % agreement)

	No new diagnosis	New accurate diagnosis	MDO implicated	Overall
First 50% of review sessions (n=1027)	383/569 (67.3%)	370/593 (62.4%)	23/116 (19.8%)	776/1027 (75.6%)
Second 50% of review sessions (n=1037)	462/574 (80.5%)	390/545 (71.6%)	12/91 (13.2%)	864/1037 (83.3%)

MDO=Missed Diagnostic Opportunity
^Split by chronological order, within each pair of reviewers

Table S4: Number of reviewer-assessed contributing factors to each MDO	
**Number of contributing factors**	**n (%)**
1	25 (28)
2	38 (43)
3	22 (25)
4	3 (4)
5	1 (1)
Total	89 (100%)
MDO=Missed Diagnostic Opportunity	

Table S5: Distribution of potential harm ratings associated with the MDOs	
**Harm rating***	**n (%)**
Unclear	4 (4.5)
No harm	5 (5.6)
Mild harm	47 (52.8)
Moderate harm	21 (23.6)
Severe harm	12 (13.5)
Total	89 (100%)
*Highest harm rating across the reviewers	

**Box S1 Structured data collection questions**
Q1. The content of earlier encounters 3 months prior to the index consultation (history, examination, differential diagnoses) suggest an alternate index diagnosis that was not considered
Q2. The content of (history, examination, differential diagnoses) suggests an alternate diagnosis that was not considered
Q3. Diagnostic testing data (laboratory, radiology, pathology etc) were suggestive of an alternate diagnosis which was not considered or misinterpreted
Q4. Alarm symptoms or 'Red flag' (i.e. features in the clinical presentation that are considered to predict serious disease) were not acted on
Q5. Clinical information present in the index consultation should have prompted additional evaluation (examination, referral, investigation, follow-up appointment)
Q6. The 'final' diagnosis was an evolution of the initial presumed index diagnosis
Q7. The clinical presentation at the index consultation was atypical given the final diagnosis
Q8. Subsequent encounters 9 months since the index consultation (out-of-hours service, Accident & Emergency department, specialist clinics, practice consultations) suggest missed diagnostic opportunities

Cheraghi-Sohi S, *et al. BMJ Qual Saf* 2021; 30:977–985. doi: 10.1136/bmjqs-2020-012594

**Box S2: Definitions used by reviewers when categorising diagnostic information from index consultations**
**1. No new diagnosis**
This rating was given unless one of three possible types of diagnosis was identified:<br>a. Those that were recorded by the diagnostician i.e. new diagnosis).<br>b. Diagnoses inferred from the record, of two forms: (i) 'Diagnosis by action' - the reviewer infers that a diagnosis has been made by the clinician during the index consultation by using both the recorded symptom and ensuing actions e.g. treatments etc; (ii) 'Diagnosis inferred by reviewer' - the reviewer infers from the information that a diagnosis should have or could have been made by the clinician in the index consultation but was not made/recorded.<br>c. Diagnoses that were made before the index consultation by another clinician but that comes to light during the index consultation
**2. Diagnosis accurate and sufficient evidence**
The diagnosis should: (i) be clear, specific and 'complete'; and (ii) be confirmed by sufficient and documented evidence from the medical record. If a patient does not consult again because of the index problem, this is not to be considered as 'evidence' that the diagnosis was correct or that it had resolved.
**3. Diagnosis accurate but insufficient evidence**
The diagnosis should: (i) be clear and accurate but less precise and/or specific compared with a rating of '1'; and (ii) have at least some documented evidence of its accuracy in the record.
**4. Diagnosis accurate but incomplete or little/no evidence**
The diagnosis should be: (i) related to the 'final' diagnosis but less precise, clear and/or specific compared with ratings of '2' or '3 above'; and/or (ii) there should be very little or no documented evidence of its accuracy in the medical record.
**5. Missed diagnostic opportunity possible**
The diagnosis should: (i) be different when compared with the final diagnosis, but the difference should be less than for ratings of '6' or '7' below; and (ii) have little or no documented, objective evidence in support of a diagnostic error. In other words, the judgment relied mainly on the reviewers' professional interpretation.
**6. Missed diagnostic opportunity likely**
The diagnosis should: (i) be substantially different from the final diagnosis; and (ii) have at least some documented evidence to support the presence of a missed diagnostic opportunity.
**7. Missed diagnostic opportunity certain**
The diagnosis should: (i) be completely different from the final diagnosis; and (ii) have compelling evidence documented to this effect.

Cheraghi-Sohi S, *et al. BMJ Qual Saf* 2021; 30:977–985. doi: 10.1136/bmjqs-2020-012594

**Box S3: Definitions used by reviewers when assessing degree of harm from missed diagnostic opportunities (MDOs)**
**1. No harm**
This rating includes 'near misses' and instances where harm was mitigated.<br>Example:<br>An index diagnosis of a 'incisional hernia' was made in a patient who had recently undergone surgical resection of colon cancer. The patient subsequently had a specialist review and CT scan that excluded this diagnosis. In this example, the specialist review and CT scan had previously been arranged to evaluate the treatment of his cancer. The diagnostic error therefore did not cause the patient any inconvenience, e.g. additional or extra investigations or harm and its impact was coded as '1'. However, if an otherwise healthy patient had been referred with an index diagnosis of 'hernia' and was subsequently found not to have this problem, the impact should have been rated as '2'.
**2. Mild harm**
This rating includes any MDO with an impact on patients that are judged to be minor or inconveniences, such as unnecessary follow-up appointments (in any setting), treatments and/or investigations. The impact of MDOs with a rating of '2' should be transient and without any residual effect.<br>Example:<br>The index diagnosis was 'iron deficiency' and the patient was commenced on supplementary iron tablets. However, subsequent haematinics and FBC were normal. The patient was informed of the results and advised to stop the treatment. The reviewer coded the diagnostic error's impact as '2'. However, if the patient had developed severe constipation resulting in an anal fissure as a result of the iron tablets, the impact of the diagnostic error should have been rated as '3'.
**3. Moderate harm**
A pragmatic test of the severity of harm is for reviewers to consider how they would have felt if the impact had been on them personally. Moderate harm includes physical or psychological distress, but should always be self-limiting in nature and completely resolve without complication over time.<br>Example:<br>The index diagnosis was 'soft tissue injury of foot' while the final diagnosis several months later (confirmed through biochemistry) was 'gout'. Further review of the record found that this patient had presented on several occasions with recurrent episodes of significant pain resulting in time off work. The reviewer therefore rated harm as '3'. However, if the patient had developed gouty tophi after years of untreated gout, then harm should have been rated as '4'.
**4. Severe harm**
Any harm with prolonged or permanent impact; e.g. preventable hospital admission/procedures/complications, disease progression, disability or death.<br>Example:<br>The index diagnosis was irritable bowel syndrome but the final diagnosis, many months later, was histological confirmed metastatic bowel cancer.
**5. Unclear**
Reviewers also have the option to select 'unclear' if there is not sufficient evidence in the record to make a judgment.

**Box S4: Examples of identified missed diagnoses, by level of harm caused**			
	**Example 1**	**Example 2**	**Example 3**
**No Harm**	**Ischaemic Heart Disease***	**Asthma**	**Folliculitis**
	Female age <50 presented with leg cramps in her calf after a long flight. The GP examined her and ruled out Deep Vein Thrombosis, with the advice to take ibuprofen. Although the correct diagnosis of the presenting symptoms was made (i.e. to rule out the suspected DVT by the patient), the GP should have also attended to other information available at the time of the index consultation, which stated that the patient had recently been admitted to hospital and told that she had had a myocardial infarction. This was then confirmed by another GP who conducted the follow-up investigations a week later and confirmed the patient as having ischaemic heart disease (IHD). The rapid pick up of the issue by another GP resulted in no harm being done to the patient.	Male age <50. Index consultation notes indicated a suspected chest infection due to chesty cough symptoms and recorded as 'likely viral'. However, the patient's history of asthma was not taken into account and in fact the recent Peak Flow Rate data available to the GP suggested an exacerbation of asthma. The patient had a history of non-compliance with their asthma medication in a later consultation had again stopped their asthma medication. Despite the misdiagnosis of a viral infection, no harm appears to have come to the patient who also continued their non-adherence to their asthma medication.	Female age <50 presented with scaly nodular lesions in the pubic area, with a follicular distribution. Notes indicated that initially the GP suspected fungal origins. Further consultations indicated issue however was most likely due to shaving. Patient was advised to stop shaving the area but the patient declined. No harm appears to have come from the initial fungal misdiagnosis.
**Mild Harm**	**Burning mouth syndrome –** lengthening of symptoms and an unnecessary referral.	**Diabetes Mellitus –** lengthening of symptoms due to untreated condition	**Florid Impetigo –** worsening of symptoms plus wrong treatment.
	Female age >50, had been seen by dentist prior to the index consultation with a sore mouth which they'd had for a month and which did not respond to antibiotics. At the index consultation thrush was suspected by the GP but the findings noted were not typical for thrush. The diagnosis of burning mouth syndrome was already suggested by the dentist's findings and the absence of clear cut signs of thrush which was the GP's diagnosis. The ultimate diagnosis of burning	Female age >50 presented with symptoms of Diabetes Mellitus and was worried that she had Diabetes Mellitus. Blood tests were performed a week later and confirmed the diagnosis but the GP did not act on the findings and the patient did not receive the diagnosis until 3 months later. An unnecessary diagnostic	Male age <50. Index consultation notes state 'likely viral' for the presenting symptoms and yet the patient was also noted to have spots. The patient returned a few days later with a rash on the left cheek which the GP then determined was "florid impetigo" that needed a prolonged course of Flucloxacillin to treat successfully.

Cheraghi-Sohi S, *et al. BMJ Qual Saf* 2021; 30:977–985. doi: 10.1136/bmjqs-2020-012594

	mouth syndrome was not surprising in view of the history and findings. Treatment with drugs for neuropathic pain such as amitriptyline are well established and pretty safe approaches that could have been tried by the GP before the unnecessary referral was made by the GP to secondary care.	delay in a patient that was symptomatic at presentation.	
**Moderate Harm**	**Prostatitis** – Lengthening of symptoms, undergone unnecessary tests, prolonged distress and unnecessary referral.	**Atypical Face Pain –** lengthening of symptoms over months and prolonged distress due to the pain.	**Gallstones** - lengthening of symptoms and prolonged distress due to the pain.
	Male age >50. At the index consultation no differential diagnosis was given to explain the symptoms of being unwell, hot and cold plus burning in the urethra. Rectal examination was not performed even though the hall mark symptoms of prostatitis had been elicited in the history. A battery of tests was ordered which picked up an abnormality that led to secondary care referral which took some time to happen. Had examination been done, then tests may not have been necessary and could have avoided secondary care referral and prolonged symptoms.	Female age >50. This patient had had long standing pains in the jaw following an extraction. Treated with multiple courses of antibiotics to no avail. In the January prior to the index consultation dental specialists indicated that the cause of the pain was atypical facial pain/complex facial pain syndrome and treatment with a Tricyclic antidepressant was recommended. This info was not acted on until many months later and the Tricyclic antidepressant did seem to help the patient.	Male age <50 presented with abdominal pain in the index consultation. However, in a visit to Accident and Emergency a few weeks prior to the index consultation, there was mention of a documented gallstone and possible pancreatitis on a USS in the letter from A & E. This was not picked up by the GP and the patient continued to have symptoms. Eventually a referral was made after some time by another GP, however the patient did not attend the review.
**Severe Harm**	**Endometrial Cancer –** disease progression with permanent impact via the severely delayed diagnosis of cancer.	**Bladder Cancer -** disease progression with permanent impact via the severely delayed diagnosis of cancer.	**Warfarin induced rectal bleeding –** the patient suffered unnecessary and invasive investigation as well as lengthening of symptoms and complications after surgery related to the error.

Cheraghi-Sohi S, *et al. BMJ Qual Saf* 2021; 30:977–985. doi: 10.1136/bmjqs-2020-012594

	Female age <50. Ultrasound Scan on pelvis showed a lesion and she was referred to secondary care with the report. The hospital booking team failed to make contact with her on several occasions. The gynaecology team did not get round to doing hysteroscopy planned in 2013 until March 15. Meanwhile she had been referred to urologist for possible stones. CT scan showed peritoneal lymph glands thoughts to be of dubious significance, referred to haematologists who surmised issues not due to lymphoma. Patient referred back to GP who referred on 2 week wait [urgent referral] to gynaecologist on receiving the results from the urologist. Then gynaecology did biopsy of endometrial polyp and found endometrial cancer resulting in Total Abdominal Hysterectomy and Oophorectomy. In summary - a series of issues. There was incomplete diagnostic evaluation by gynaecology when they did not follow through on the endometrial polyps, cancelled appointments and a failure to organise hysteroscopy. A repeated failure of attempts to contact patient to arrange appointments and from the GP for not pursuing these issues.	Female age >50. Patient was referred for haematuria but then did not attend the clinic. Patient then seen again by GP about unrelated issues and with urinary issues. Patient was referred again, cystoscopy requested which she did not attend. Patient had CT scan which showed hydronephrosis, she then eventually did have cystoscopy which confirmed cancer. She did not accept treatment initially preferring to have herbal remedies and also travelled abroad for some treatments. She missed subsequent oncology appointments and continued herbal treatments. She had capacity to decide and language issues addressed, eventually did have chemotherapy months later.	Male >50. This man has been investigated for rectal bleeding which in the notes the GP had written to have "started after starting warfarin" with no abnormality on colonoscopy. Bleeding was then attributed by the GP to piles. Haematology investigations were conducted for Iron deficiency and high reticulocytes and pancytopenia. The GP did not consider the effects of his warfarin as the cause of the bleeding. Patient went on to have prolonged bleeding after a Carpal Tunnel Syndrome operation too.
**Unclear**	**Drug Misuse**	**Chronic Obstructive Pulmonary Disease**	**Depression**
	Male age >50. The patient had recently attended a Transient Ischaemic Attack clinic. The clinic had recorded the issue and made the diagnosis of drug misuse. It was also mentioned on two previous occasions in prior consultations as recorded in the notes. The clinic letter and other diagnostic information (prior notes) relating to the drug misuse, were available to the GP at the index consultation but it was not picked up or coded. It is unclear why the drug use not picked up or addressed. Harm was not clear because no further	Male age >50. GP notes suggest a chest infection, however the GP did not seem to take into account that the patient had previously had numerous other "chest infections" in the notes prior to the index consultation. The patient subsequently presented to another GP with persistent respiratory symptoms, and was referred through to the practice nurse for	Female age <50 presented with symptoms of being tired all the time and with psychological disturbances. Her bloods were OK. The history obtained outlined multiple traumatic issues in her life including the patient having refugee status, and her husband being imprisoned and tortured. A referral for psychiatric assessment

Cheraghi-Sohi S, *et al. BMJ Qual Saf* 2021; 30:977–985. doi: 10.1136/bmjqs-2020-012594

	mention of drug use and it was therefore unclear if he continued using and whether this may have had effects on his blood pressure which was raised, in later consultations. The details of drug use were not explored and whether there were links if any to his Ischaemic Heart Disease/Cerebrovascular Accident symptoms.	spirometry, which showed that he had COPD one month later. The patient was then prescribed an inhaler. It is unclear what harm the patient came to from this short delayed diagnosis as the patient did not re-attend in the interim, condition appeared not to deteriorate and the correct treatment was prescribed swiftly upon the diagnosis being made.	was discussed in the index consultation indicating that the GP suspected further psychological issues to her current depressive state. No referral or follow up occurred however for the patient who also didn't consult again with this issue so it is unclear as to the harm outcomes for this patient.
*Missed diagnosis and main harms caused (where relevant)			

Figure S1 Conceptual Model of Missed Opportunities in Diagnosis (MDOs). MDOs are events falling into areas A and B of the model. Source: Singh et al[1]

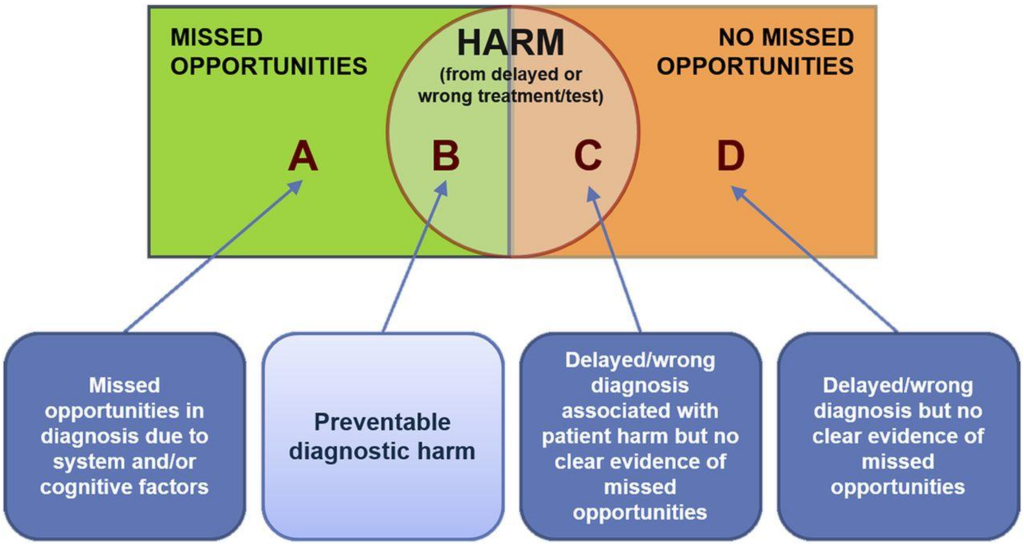

Cheraghi-Sohi S, *et al. BMJ Qual Saf* 2021; 30:977–985. doi: 10.1136/bmjqs-2020-012594

Figure S2: Overview of review process at each practice

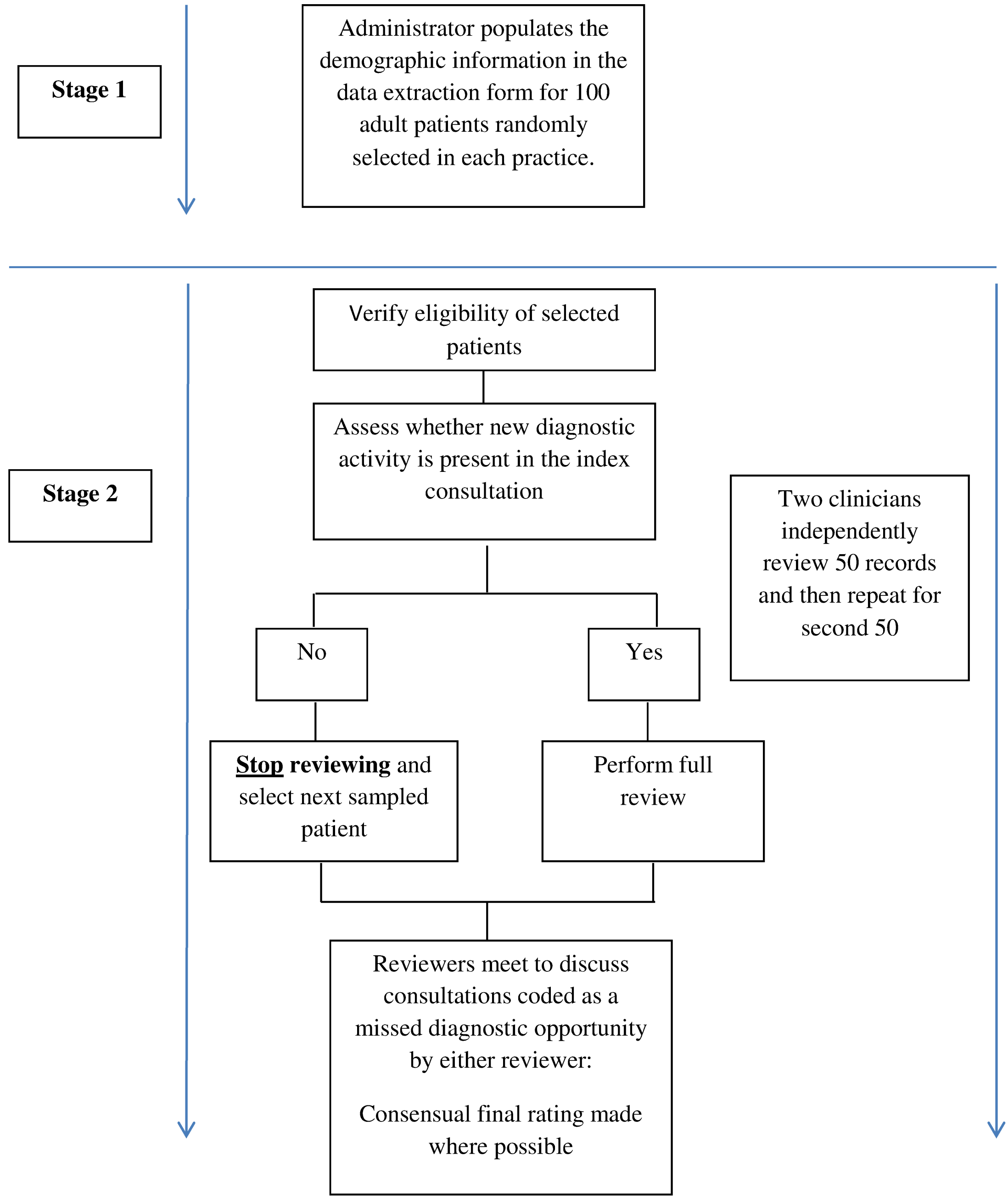

Cheraghi-Sohi S, *et al. BMJ Qual Saf* 2021; 30:977–985. doi: 10.1136/bmjqs-2020-012594

REFERENCES

1. Singh H. Editorial: Helping Health Care Organizations to Define Diagnostic Errors as Missed Opportunities in Diagnosis. *The Joint Commission Journal on Quality and Patient Safety* 2014;40(3):99-AP1. doi: https://doi.org/10.1016/S1553-7250(14)40012-6
